# An exploratory study of two-dimensional shear-wave elastography in the diagnosis of acute compartment syndrome

**DOI:** 10.1186/s12893-021-01420-y

**Published:** 2021-12-15

**Authors:** Jun Zhang, Wanfu Zhang, Huihui Zhou, Lin Sang, Lina Liu, Yuanyuan Sun, Xue Gong, Hao Guan, Ming Yu

**Affiliations:** 1 The Department of Ultrasound, Xijing Hospital, The Fourth Military Medical University, No. 127 Changle West Rd, Xi’an, 710032 Shaanxi China; 2 The Department of Burns and Cutaneous Surgery, Xijing Hospital, The Fourth Military Medical University, No. 127 Changle West Rd, Xi’an, 710032 Shaanxi China

**Keywords:** Ultrasound 2D shear-wave elastography, Acute compartment syndrome, Elasticity value of muscle, Noninvasive measurement

## Abstract

**Background:**

Two-dimensional shear-wave elastography (2D-SWE) is an ultrasound elastography technique that uses shear waves to quantitatively measure tissue stiffness and it has recently been developed as a safe, real-time, and noninvasive imaging technique. The purpose of this study was to investigate the value of 2D-SWE in the diagnosis and treatment of acute compartment syndrome (ACS).

**Methods:**

2D-SWE was used to measure the elasticity values of the main muscles in the superficial compartments of the calf in 212 healthy volunteers, and the difference in the muscle elasticity values between different gender and age groups were analyzed. Nine patients with clinical suspicion of ACS were included in this study and 2D-SWE was used to measure the elasticity values of the muscles on the affected and unaffected sides, and a comparative analysis was performed.

**Results:**

The mean elasticity values of the tibialis anterior (TA), peroneus longus (PL), and gastrocnemius medialis (GA) muscles in the relaxed state of the 212 healthy volunteers were 25.4 ± 3.2 kPa, 15.7 ± 1.5 kPa, and 12.1 ± 2.1 kPa, respectively. No statistically significant differences was observed in the elasticity values of the same muscle under the state of relaxation in different gender and age groups (*p* > 0.05). A statistically significant difference in the elasticity values of the muscle between the affected and unaffected sides in the fasciotomy group (*p* < 0.05, n = 5) was observed. In contrast, no difference in the elasticity values of the muscle between the affected and unaffected sides in the conservative group (*p* > 0.05, n = 4) was observed. There was a statistically significant difference in the elasticity values of the muscle on the affected side in the two treatment groups (*p* < 0.05).

**Conclusions:**

When the ACS occurs, the muscle elasticity of the affected limb increases significantly. 2D-SWE is expected to be a new noninvasive technique for the assessment of ACS and may provide a potential basis for clinical diagnosis and treatment.

## Background

Acute compartment syndrome (ACS) is a clinical surgical emergency that refers to an increase in intra-compartmental pressure (ICP) caused by various reasons, such as fractures, crush injuries, and thermal injuries, resulting in a decrease in perfusion pressure, leading to hypoxemia of the tissues [1]. Decreased tissue perfusion can lead to irreversible necrosis that may result in limb dysfunction, amputation, and even death. The forearm and the lower leg, especially the latter, are more prone to develop compartment syndrome owing to their double-bone structure, and thick, tough, and inelastic interosseous membrane and fascia [2]. The lower leg is the most common location of ACS, which consists of the following four compartments: anterior, lateral, superficial posterior, and deep posterior, with the anterior and lateral compartments most frequently affected, especially the anterior compartment. The tibialis anterior (TA), peroneus longus (PL), and gastrocnemius (GA) are the main muscles in the first three compartments, respectively [3]. Currently, the diagnosis of ACS mainly depends on the clinical manifestations and measurement of the ICP by some invasive methods, such as the Whitesides needle manometer, a slit or wick catheter [4]. However, the judgment of clinical manifestations is subjective and delayed. To date, no definite pressure threshold value that can be used as an indication of early incision decompression is available [5]. Moreover, the measurement methods of ICP are invasive, increasing the risk of infection and patient’s symptoms [6–8]. Therefore, we wish to find a real-time, noninvasive, safe, and accurate assessment method for the diagnosis and treatment of ACS.

Ultrasonography, one of several main clinical imaging techniques, has distinct strengths for the evaluation of superficial soft-tissue diseases. Two-dimensional shear-wave elastography (2D-SWE) is an ultrasound elastography technique that uses shear waves to quantitatively measure tissue stiffness, and it has recently been developed as a safe, real-time, and noninvasive imaging technique [9]. By quantifying mechanical and elastic tissue properties, SWE complements the diagnosis information obtained via gray-scale US and color Doppler US. As a transverse wave, the shear wave occurs in an elastic medium that is subject to a periodic shear force, and shear is defined as change in the shape of a substance layer without volume change, produced by a pair of equal forces working in opposite directions along the two opposed sides of the layer [10]. To characterize tissue stiffness with SWE does not require knowledge of applied stress [11, 12].

Currently, 2D-SWE is relatively mature and widely used in the examination and evaluation of the superficial organs (such as thyroid, breast, and skin, among others) [13–15] and abdominal organs (such as liver, spleen, and kidney, among others) [16–18]. The application and efficacy of 2D-SWE in the evaluation of several traumatic and pathological conditions of various musculoskeletal soft tissues, including the muscles [19–22], tendons [23, 24], ligaments [25], and nerves [26–28] have been reported. Quantitative elastography can not only greatly help by providing a parameter that can influence the diagnosis, but also providing a way to monitor the effectiveness of a treatment by quantifying the changes in the mechanical state of the muscle [28]. The ICP was increasing when ACS occurred, and Steinberg observed a strong correlation between the stiffness of the muscle compartment and the ICP [29]. The measurement of the elasticity value of muscle tissue has been suggested as one of the most promising techniques to detect and objectify an ACS [30]. However, the utility of 2D-SWE for diagnosis of ACS is still unclear, mainly owning to the lack of standardization of the elasticity values of muscles in healthy subjects.

The aim of our study was to evaluate the muscles in the fascia compartment of healthy volunteers and suspected-ACS patients by multimodal ultrasound (two-dimensional, color Doppler, and 2D-SWE), and to explore whether multimodal ultrasound (especially 2D-SWE) could be used as an effective inspection method for the diagnosis and treatment of ACS.

## Methods

### Research participants

We performed a prospective analysis of nine patients with limb swelling suspected of acute compartment syndrome and 212 healthy volunteers who were included in this study with their consent (Tables 1, 2, 3). The inclusion criteria of the volunteers were as follows: age range: 20–70 years, body mass index (BMI) in the normal range (18.5–23.9, Chinese reference standard for BMI), and no history of musculoskeletal system diseases or trauma or neurological diseases. The volunteers were divided into three groups according to the World Health Organization (WHO) classification: group I, young people (age ≤ 44 years); group II, middle age (age 45–59 years); group III, elderly (age 60–89 years) [31]. All participants provided oral informed consents. All the procedures described in this study were conducted in accordance with the Declaration of Helsinki and International Council for Harmonisation/Good Clinical Practice Guidelines approved by the institutional review board.

**Table 1:** Mean elasticity values for TA, PL, GA in the gender groups (n = 212, mean ± standard deviation)

Muscles/elasticity(kPa)	Total (n = 212)	Male (n = 100)	Female (n = 112)	*p *value*
Tibialis anterior	25.4 ± 3.2	25.7 ± 3.4	25.2 ± 3.0	0.232
Peroneus longus	15.7 ± 1.5	15.9 ± 1.7	15.6 ± 1.2	0.157
Gastrocnemius medialis	12.1 ± 2.1	12.3 ± 1.9	11.9 ± 2.2	0.149

^*^The independent samples t-tests were performed and a *p*-value < 0.05 was considered to indicate statistical significance

**Table 2:** Mean elasticity values for TA, PL, GA in different age groups (n = 212, mean ± standard deviation)

Muscles/elasticity(kPa)	≤ 44 y (n = 104)	45–59 y (n = 84)	≥ 60 y (n = 24)	*p *value*
Tibialis anterior	25.1 ± 3.3	25.8 ± 3.2	25.2 ± 2.9	0.300
Peroneus longus	15.4 ± 1.8	15.5 ± 1.6	15.4 ± 1.6	0.962
Gastrocnemius medialis	12.1 ± 2.3	12.0 ± 2.0	12.3 ± 1.8	0.870

^*^The one-way analysis of variance were performed and a *p*-value < 0.05 was considered to indicate statistical significance

**Table 3:** The table summarizes the patients with acute compartment syndrome following fasciotomy (n = 5) and conservative treatment (n = 4)

Patient	Gender	Age(y)	Body mass index(kg/m^2^)	History	Conventional US of the affected side	2D SWE (kPa, mean ± standard deviation)		Ratio of affected to unaffected side	Treatment
						Affected side	Unaffected side		
**1**	M	47	23.56	Empyrosis	The echo of the muscle layer in the right calf was disorganized, the continuity of local muscle texture was interrupted, and no obvious blood flow signal was detected inside	268.8 ± 18.9	27.6 ± 1.5	9.74	Fasciotomy
**2**	M	24	22.53	Empyrosis	The skin and subcutaneous adipose layer of the affected side is oedematous, thickened, the echo of muscular layer has not seen obvious abnormality	23.5 ± 2.1	22.4 ± 1.9	1.05	Conservative treatment
**3**	M	48	23.32	Empyrosis		48.5 ± 1.5	24.2 ± 3.2	2.00	Prophylactic fasciotomy
**4**	M	35	24.68	Aortic dissecting aneurysm	The tibialis anterior of affected side is thickened with decreased echo than the heathy side	173.8 ± 16.7	31.6 ± 2.8	5.50	Fasciotomy
**5**	M	61	23.89	Aortic dissecting aneurysm		128.1 ± 23.1	32.1 ± 2.1	3.99	Fasciotomy
**6**	M	34	23.35	Direct trauma	The hematoma in the right gastrocnemius medialis abdominal space was about 11.0 × 1.2 × 2.5 cm	30.9 ± 2.7	27.9 ± 2.2	1.11	Conservative treatment
**7**	M	37	22.48	Aortic dissecting aneurysm	Subcutaneous adipose layer of the right calf is oedematous, thickened, the echo of muscular layer has not seen obvious abnormality	26.3 ± 2.6	22.2 ± 1.9	1.18	Conservative treatment
**8**	F	68	19.23	Electrical injury	The subcutaneous adipose layer and the pronator teres muscle were edema and thickened compared with the contralateral side, with enhanced echo and increased color blood flow signals	55.6 ± 2.4	18.1 ± 1.9	3.07	Conservative treatment
**9**	F	66	19.53	After percutaneous right femoral artery covered stent implantation	The echo of the muscle layer in the right calf was enhanced and disorganized, the continuity of local muscle texture was interrupted, and no obvious blood flow signal was detected inside	242.4 ± 44.1	30.4 ± 4.2	7.97	Fasciotomy

### Multimodal ultrasound imaging (two-dimensional, color Doppler, 2D-SWE)

In this study, all imaging procedures (two-dimensional, color Doppler, and 2D-SWE) were performed using an L15-4 high-frequency linear array probe (4–15 MHz) on a Supersonic Imagine ultrasound system (Aixplorer, Supersonic Imagine, Aix-en-Provence, France) by a sonologist with 3 years of work experience, and all sonograms were reviewed by a senior professional post sonologist with 20 years of work experience. The muscles selected for observation were scanned using two-dimensional and color Doppler ultrasound in transverse and longitudinal sections to assess their echo characteristics and blood flow information, followed by shear-wave elastography (SWE) in longitudinal sections. We used SWE standard mode with the tissue Young's modulus values reported in kilopascals (kPA). The stiffness of muscles was displayed inside the frame by color change from blue (soft) to red (hard) depending on the Young's modulus.

Basic information of the nine suspected-ACS patients with swollen limbs are shown in Table 3 (patients were numbered from one to nine in chronological order of admission). Among them, eight patients had swelling in the calves and one patient had swelling in the wrist (Hereinafter referred to as “affected side”). Therefore, we selected the corresponding muscles as the objects of multimodal ultrasound imaging (TA, GA, pronator teres) before treatment. In addition, the same muscles on the healthy sides (Hereinafter referred to as “unaffected side”) were scanned as controls.

Another objective of our study was to observe the normal range of elasticity values of the main muscles in each compartment at the resting state. The imaging depth of deep posterior compartment of the leg was a limitation; hence, for the volunteers, we evaluated TA, PL, and GA muscles in the anterior, lateral, superficial posterior compartments by multimodal ultrasound imaging, and observed whether gender and age were independent effect factors.

### Protocols of 2D-SWE

All participants were in the supine position, their limbs laid naturally on the examination bed, and the muscles were in relaxed state. The central location of the target muscle was selected for the measurement, away from the epimysium. The sonologist was asked to scan a given location using minimized transducer pressure. The elastography function was enabled when the gray-scale image at the long axis section was clear, a suitable region of interest (ROI) based on the center of the muscle and clear gray-scale image was chosen. The color reflecting the Young’s modulus value was evenly filled and stabilized for at least 5 s before freezing the image for measurement. Subsequently, circles with diameters of 10 mm, 8 mm or 5 mm were generated at the center of the range depending on the muscle size. Subsequently, the Young’s modulus was automatically calculated as the muscle elasticity values. The Young’s modulus for each muscle was measured five times, and the three mean values with the lowest coefficient of variation over the circle were used in this study.

### Statistical analysis

The SPSS software (version 20.1, SPSS, IBM) was used for data analysis. Continuous variables are expressed as mean ± standard deviation. Normality of the distribution of data were verified by using the Shapiro–Wilk test. The independent samples t-test was used to compare the elasticity values of muscles in men and women. The one-way analysis of variance was used to compare the measurement data of different age groups. Elasticity values of the affected and unaffected sides was analyzed by paired samples t-test, and the independent two-sample mean difference t-test was used to compare the measurement data of different treatment groups. A *p*-value < 0.05 was considered statistically significant.

## Results

Among the 212 volunteers, 100 were male participants with an average age of 44.5 ± 14.8 years, and 112 were female participants with an average age of 42.6 ± 12.4 years. The other nine patients were highly suspected of ACS by clinicians according to their clinical symptoms (such as worsening pain that is out of proportion and increasing analgesic requirements, swelling, paresthesia or anxiety, agitation), their average age was 46.7 ± 14.7 years (range: 24–68 years).

### Analysis of differences in muscle elasticity values between genders

**Fig. 1:**
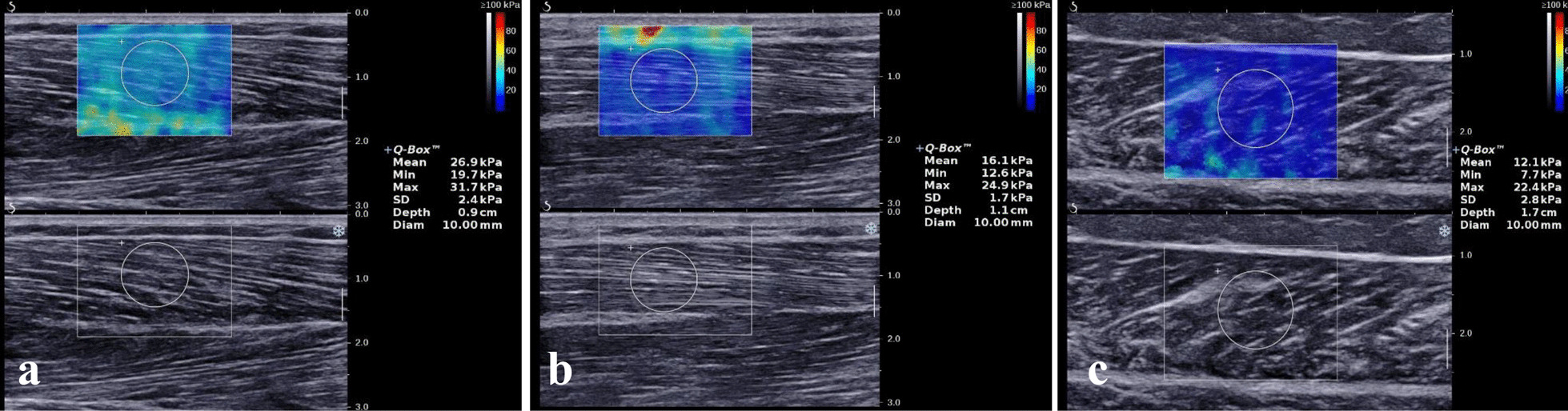
The elasticity values of major muscles (**a** Tibialis Anterior, **b** Peroneus Longus, and **c** Gastrocnemius medialis) in each compartment of the calf of a 42 years old volunteer measured by 2D-SWE

Statistical analysis was conducted on the elasticity values of major muscles (TA, PL, and GA) in each compartment of the calves of 212 volunteers. The results showed that the mean elasticity values of TA, PL, and GA muscles in the relaxed state were 25.4 ± 3.2 kPa, 15.7 ± 1.5 kPa, and 12.1 ± 2.1 kPa, respectively. The mean elasticity values of the TA, PL, and GA for men and women were 25.7 ± 3.4 kPa, 15.9 ± 1.7 kPa, 12.3 ± 1.9 kPa, and 25.2 ± 3.0 kPa, 15.6 ± 1.2 kPa, and 11.9 ± 2.2 kPa, respectively (*p*-values were 0.232, 0.157, and 0.149, respectively). No statistically significant differences in the elasticity values of the same muscle under the state of relaxation in men and women were observed (*p* > 0.05, Table 1; Fig. 1).

### Analysis of differences in muscle elasticity values between different age groups

We divided 212 volunteers into three groups by age, and measured the elasticity values of TA, PL, and GA muscles in each group. Among them, 104 volunteers were ≤ 44 years old, 84 were 45–59 years old, and 24 were ≥ 60 years old. The elasticity values of the TA, PL, and GA muscles in different age groups were shown in Table 2 (*p*-values were 0.300, 0.962, and 0.870, respectively). The statistical analysis results revealed that there were no statistically significant differences in the elasticity values of the same muscle in the state of relaxation in different age groups (*p* > 0.05).

### Analysis of elasticity values of the muscles in the relevant compartments of nine patients with clinical suspicion of ACS

Among the nine suspected-ACS patients (seven men, two women), three patients were admitted for aortic dissection aneurysm, three patients were admitted for burns of different degrees, one patient was admitted for intramuscular hematoma caused by trauma, one patient was injured by electric shock, and one patient was admitted after percutaneous right femoral artery covered stent implantation.

**Fig. 2:**
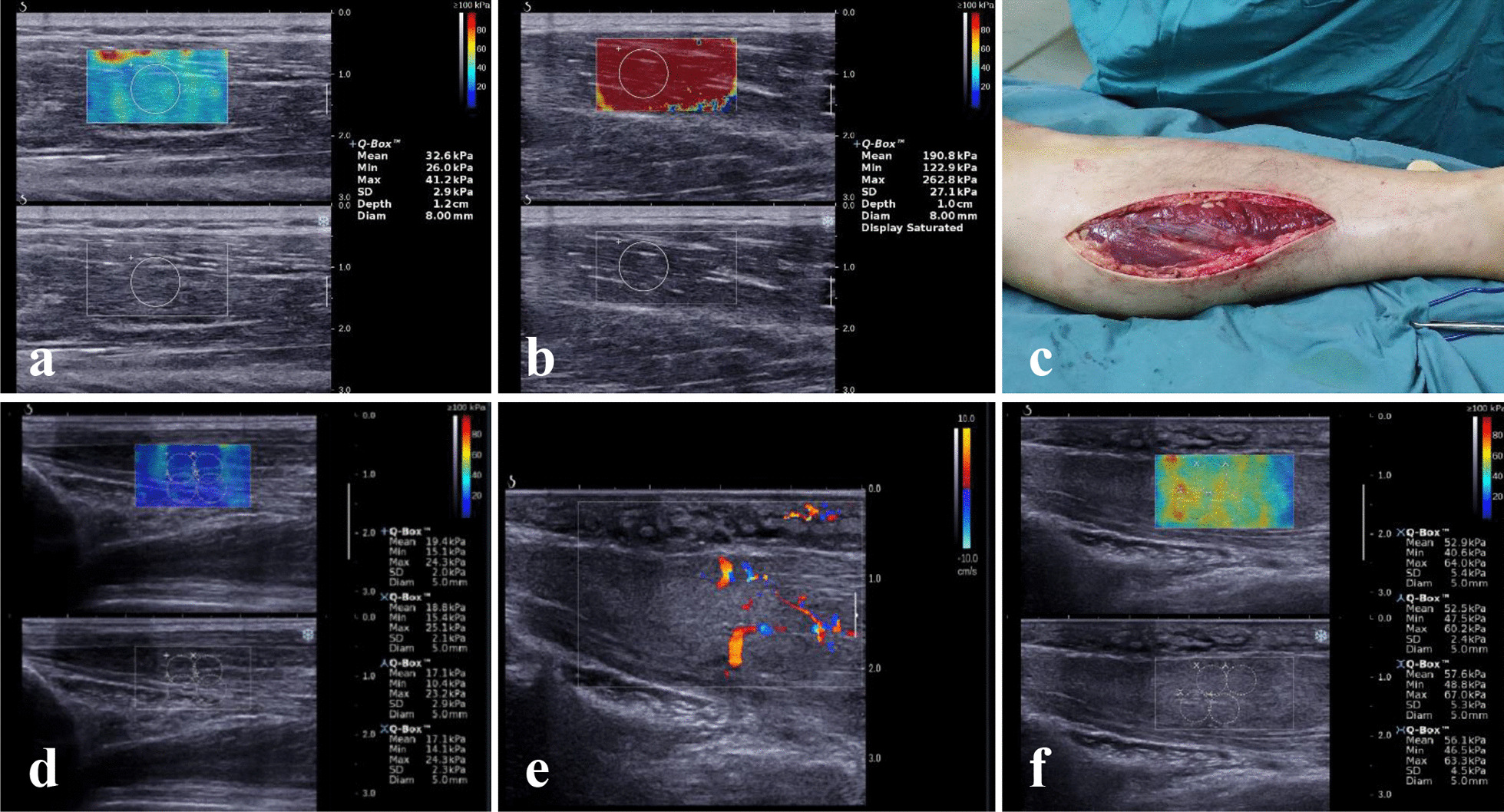
The images of 2D-SWE and fasciotomy in case 4 of Table 3 (a–c): **a** The elasticity value of TA in the unaffected side. **b** The elasticity value of TA in the affected side. **c** The patient underwent a fasciotomy for decompression. The images of case 8 of Table 3 (**d**–**e**): **d** The elasticity values of pronator teres in the unaffected side. **e** 2D and CDFI images showed that the subcutaneous adipose layer and the pronator teres muscle were edema and thickened with enhanced echo and increased color blood flow signals. **f** The elasticity values of pronator teres in the affected side

Conventional ultrasound examination and ultrasound elastography were performed (Fig. 2) for these patients. We found that the patients number 1, 4, 5, and 9 not only experienced thickening of the subcutaneous fat layer of the affected limbs, but also experienced thickening of the muscle layer to varying degrees with decreased muscle echo intensity, and the continuity of muscle texture disappeared in some patients. In addition, the elasticity value of the muscle on the affected side was significantly higher than that of the same muscle on the healthy side (the minimum ratio of affected to unaffected side was 3.99). In the other patients (number 2, 3, 6, 7, and 8), two-dimensional ultrasound imaging revealed edema and thickening of the subcutaneous adipose layer at corresponding sites, while no obvious abnormalities were found in the echo and thickness of the muscular layer. Furthermore, no significant increase or only a relatively small increase in the elasticity values was measured by 2D-SWE of the muscles on the affected side than on the healthy side (or compared to the healthy volunteers) (Table 3).

Finally, five patients (number 1, 3, 4, 5, and 9) were treated with surgical fasciotomy for decompression, and four patients (number 2, 6, 7, and 8) were treated conservatively (non-surgical treatment). In patients number 1, 4, 5, and 9, obvious muscle necrosis was found after surgical fasciotomy. In patient number 3, the clinician performed a prophylactic incision based on the progression of the disease, and the muscle was found intact during the surgery. All four patients received conservative treatment with satisfactory results, and the limb swelling gradually disappeared and returned to normal.

The elasticity values of the muscles measured by 2D-SWE in patients who underwent different types of treatments are shown in Table 4. A statistically significant difference in the elasticity values of the muscle on the affected and unaffected sides in the fasciotomy group (*p* < 0.05, n = 5) was observed. In contrast, there was no difference in the elasticity values of the muscle on the affected and unaffected sides in the conservative group (*p* > 0.05, n = 4). There was a statistically significant difference in the elasticity values of the muscle on the affected side in the two groups (*p* < 0.05).

**Table 4:** Comparison of the elasticity values by using 2D-SWE according to the type of treatment

Type of treatment	2D-SWE (kPa, mean ± standard deviation)	*p *value
Fasciotomy (n = 5)		0.019^(1)^
Affected side	172.3 ± 72.4	0.022^(2)^
Unaffected side	29.2 ± 2.7	
Conservative treatment (n = 4)		
Affected side	34.1 ± 12.7	0.282^(2)^
Unaffected side	22.7 ± 3.5	

^(1)^Difference between intergroup (Affected side, Fasciotomy VS conservative treatment), the independent two-sample mean difference t-test was performed

^(2)^Difference between intragroup (Affected side VS unaffected side), the paired sample t-test was perform; A *p*-value < 0.05 was considered statistically significant

## Discussion

The diagnosis of ACS is challenging, particularly in specific groups, such as children and patients with consciousness disorder who fail to express their chief complaints [32], and to date, the only effective treatment is surgical fasciotomy. Although ACS is generally considered as an “orthopedic emergency,” frequent delays exist in the time from initial assessment to diagnosis and in the time from diagnosis to surgery in patients with ACS [33]. Delayed fasciotomy is the most important factor contributing to a poor prognosis, with potentially catastrophic consequences, such as permanent sensory impairment, ischemic contracture, muscle dysfunction, amputation, and even death. Hence, early and timely diagnosis and prediction of the incidence of ACS are conducive for prevention and treatment, and protection of the limb function for the patients.

Currently, the diagnosis of ACS is based on physical examination and repeated needle sticks over a short time frame to measure ICP. A method for the accurate and reproducible diagnosis of ACS, especially for the patients with obtunded, polytrauma, or consciousness disturbance, is yet to be developed [34]. The symptoms and signs indicative of ACS have been defined as the 5Ps: pain out of proportion, pallor, paresthesias, paralysis, and pulselessness. Moreover, paralysis, and pulselessness, pallor, and reduced capillary refill time are the late clinical signs. Although previous studies have indicated that combining clinical symptoms and signs raises the sensitivity for diagnosing ACS, and an association of clinical findings with compartment syndrome seems evident, the predictive value of the clinical findings for the diagnosis of compartment syndrome is yet to be delineated [35]. It is not enough to rely solely on the clinical manifestations, especially when patients with impaired consciousness and children are involved [36].

Pressure measurement is the standard method for the diagnosis of ACS, but it is an invasive testing technique, and the process is painful and difficult to repeat. Moreover, most of these invasive methods can only perform “point detection” on the tissue pressure in the osteofascial compartment, which cannot fully reflect the pressure in multiple osteofascial compartments, and it is not convenient for continuous observation of the changes of ICP. The accuracy and reliability of these invasive methods has been questioned [37, 38]. Therefore, it is not practical in clinical practice, and most clinicians do not adopt this method, but make diagnosis based on clinical experience and 5P manifestations. There is an urgent clinical need for a less invasive or noninvasive, reliable, safe, and accurate assessment method for the early prevention or treatment of ACS before irreversible muscle ischemia occurs.

Ultrasonography has been widely applied in the diagnosis of musculoskeletal diseases, and some researchers, both at home and abroad, have tried to apply ultrasound imaging in the evaluation of compartment syndrome [11, 39, 40]. Ultrasonic imaging can clearly show the room form and structure of the crus fascia, and when ACS occurs, the two-dimensional images of gray-scale sonography, cross-sectional area of the anterior compartment of the lower leg, pretibial artery diameter, and blood flow dynamics parameters are characteristic changes, and these characteristic changes have good correlation with the fascia indoor pressure and clinical treatment [41]. Gershuni et al. used ultrasound to measure the anterior compartment thickness of the lower leg to evaluate the osteofascial pressure [42], and Mühlbacher et al. investigated the feasibility of noninvasive ultrasound-guided angle measurement as a surrogate of increased pressure in a model of ACS [43]. 2D-SWE is a technique to objectively quantify the elasticity value of the tissues, and has substantial potential for characterizing different soft tissues for clinical diagnosis [12]. It shows high reproducibility when used in the assessment of the liver, breast, and thyroid, however, its application in musculoskeletal diseases still needs further study. 2D-SWE can dynamically quantify and evaluate the real-time changes in muscle elasticity value, and obtain the changes in passive (static) muscle stiffness, dynamic muscle stiffness, and active muscle stiffness under various states by evaluating the viscoelastic characteristics of the muscle [44–46].

ACS could result in an increase in the internal pressure of the compartment and the corresponding increase in muscle stiffness in the compartment. We can indirectly reflect the intra-compartment pressure by measuring muscle stiffness in the compartment through 2D-SWE. Currently, an increasing number of studies are being conducted on the application of 2D-SWE to evaluate skeletal muscle stiffness in different parts of the healthy population, and increasing methodological studies on measurement of skeletal muscle stiffness, which fully reflects the feasibility of 2D-SWE in evaluating muscle stiffness, are being conducted [11, 40].

In this study, we measured the elasticity values of major muscles in each calf’s compartment of 212 healthy volunteers under the relaxed state by 2D-SWE, and found that there were no significant differences in the elasticity values of the same muscle in volunteers of different ages and genders under the relaxed state, which provided a basic reference value for our further study. Therefore, we believe that 2D-SWE technology is expected to be a new and effective way to diagnose ACS. In our study, 2D-SWE technique was used to measure the elasticity value of the same muscle on the affected and unaffected sides of the patients who were suspected of ACS, and comparison analysis was conducted. We found that the elasticity value was increased in different degrees on the affected side than on the unaffected side, and the increase of elasticity values were consistent with the patient’s clinical manifestations and the tension of corresponding parts. Therefore, we hypothesized that the elasticity values of muscles in compartment measured by 2D-SWE could indirectly reflect the intra-compartment pressure.

Based on our findings, we suggest that 2D-SWE offers the following advantages over invasive methods such as pressure measurement and subjective assessments based on clinical manifestations: (1) The elasticity value of muscle in compartment was measured by 2D-SWE noninvasively; (2) It can be used to measure the elasticity values of different muscles in different compartments, as well as to measure different parts of the same muscle conveniently; (3)The measurement by 2D-SWE can be repeated and is easy to operate without pain; (4)2D-SWE can also be used for dynamic monitoring and evaluation, providing real-time two-dimensional sonogram of muscles and changes in elasticity value to clinicians, and most importantly the basis evidence for treatment mode selection and adjustment.

This exploratory study has several limitations. First, we assumed that muscle elasticity values at different time periods during the occurrence of ACS could be obtained through massive data analysis, so as to provide clinicians with warning elasticity values that would require surgical fasciotomy. But only nine cases were included in this study, more cases and data need to be collected for further improvement. Second, almost all the patients under observation were critical patients in the intensive care unit, which means that it is a tough road of diverting them to the ultrasound department for examination. Real-time monitoring required us to repeatedly enter the intensive care unit with ultrasonic instruments, which is time-consuming and laborious. We hope that with the continuous development and progress of technology, portable bedside ultrasonic instruments could be equipped with 2D-SWE function, making our operation more convenient. In summary, real-time shear-wave ultrasound elastography has a great potential for the evaluation of the mechanical properties of the skeletal muscles. However, compared with clinical evaluation and other methods, choosing various measurement parameters and standardizing the inspection procedure are still issues that need further assessment.

## Conclusions

Two-dimensional shear-wave ultrasound elastography can not only visually observe muscle damage and related blood flow information in the compartment by using two-dimensional and color Doppler imaging, but can also noninvasively measure the elasticity values of muscles, which can indirectly reflect the intra-compartment pressure. When the ACS occurs, the muscle stiffness of the affected limb increases significantly. 2D-SWE is expected to be a new technique to evaluate ACS which may provide a potential basis for the clinical diagnosis and treatment of ACS and could be used as an auxiliary diagnostic method for ACS. In addition, further studies on muscle SWE standardization in different compartments are needed.

## Data Availability

The datasets used and analyzed during the current study are available from the corresponding author on reasonable request.
